# Refractive and corneal astigmatism in Chinese 4–15 years old children: prevalence and risk factors

**DOI:** 10.1186/s12886-023-03201-y

**Published:** 2023-11-10

**Authors:** Luoli Zhang, Li Zeng, Yuhao Ye, Zhe Zhang, Fang Liu, Yiyong Xian, Yang Shen, Ling Sun, Ye Xu, Ke Zheng, Xingtao Zhou, Jing Zhao

**Affiliations:** 1 Department of Ophthalmology, Eye and ENT Hospital of Fudan University, 200031 Shanghai, China; 2 National Health Commission Key Lab of Myopia (Fudan University), 200031 Shanghai, China; 3 Shanghai Research Center of Ophthalmology and Optometry, 200031 Shanghai, China; 4 Shanghai Engineering Research Center of Laser and Autostereoscopic 3D for Vision Care, 200031 Shanghai, China

**Keywords:** Astigmatism, Corneal astigmatism, Children, Prevalence, Risk factor

## Abstract

**Background:**

To investigate the prevalence and risk factors of refractive astigmatism (RA) and corneal astigmatism (CA) in preschool children and school-aged children in Shanghai, China.

**Methods:**

In this school-based, cross-sectional study, 4–15 years old children across three learning stages of kindergarten, primary school, and junior high school underwent noncycloplegic autorefraction and completed comprehensive questionnaires involving time spent on daily homework and outdoor activities. Data from the right eyes were analysed.

**Results:**

Overall, 7084 children (mean ± standard deviation (SD) of age: 8.08 ± 3.11 years) were included, and the prevalence rates of RA/CA ( ≤ − 1.0 D) in children were 15.8%/64% in kindergartens, 16.5%/65% in primary schools, and 32.8%/76.9% in junior high schools. The magnitude and prevalence of RA and CA all increased with age or with learning stage (all *P* < 0.001). The presence of RA was associated with more myopic spherical power (odds ratio (OR) 0.956, *P* = 0.021), junior high school (OR 1.973, *P* < 0.001), longer homework time on weekdays (OR 1.074, *P* = 0.029), and shorter outdoor activity time on weekends (odds ratio 0.929, *P* = 0.013).

**Conclusion:**

In the wide age range of 4 to 15 years, the magnitude and prevalence of RA and CA increased with the learning stage, and these increases mainly began at the primary school stage. Factors, including longer homework time and shorter outdoor time were correlated with the presence of RA.

**Supplementary Information:**

The online version contains supplementary material available at 10.1186/s12886-023-03201-y.

## Background

As a kind of common refractive errors, astigmatism is a clinical and public health issue [1]. It produces multiple focal points or lines in the eye and causes blurred retinal image, leading to blurred vision. Therefore, uncorrected astigmatism may affect visual development in children [2] and increase the risk of refractive amblyopia [3, 4] and myopia [5, 6]. Currently, the following two components of astigmatism can be measured and calculated: refractive astigmatism (RA) and corneal astigmatism (CA). RA refers to total astigmatism, and it includes CA, which is calculated using an equivalent refractive index. Several studies have reported the divergent prevalence of RA and CA in preschool and school-age children across the world. In China, the prevalence of RA and CA (≥ 1.0 D) were reported to be 17.4% and 52.8%, respectively in 12-year-olds in Anyang city [7]. Some studies showed 32.9% of RA (≥ 1.0 D) in children aged 7–11 years in Taiwan [8] and 12.7% of RA (≥ 1.0 D) in 4–6-year-old children in Shanghai [9]. A study showed the prevalence of RA and CA (≥ 1.0 D) were 6.7% and 26.6% in 12-year-old children [10] in Australia. In the United States, an overall prevalence of 28.4% was reported in children aged 5–17 years from four ethnic groups [11]. One study [12] conducted in Northern Ireland showed that the prevalence of RA and CA (≥ 1.0 D) were 24% and 29% in white children aged 6–7 years, and were 20% and 25% in children aged 12–13 years.

In recent years, the academic load has gradually increased and varied at different learning stages, and the prevalence of myopia has been reported to increase with the advancement of learning stage [13]. Genetic and environmental factors have also been shown to affect astigmatism development [1, 14]. With the development of children’s eyeball during childhood, as well as the increasing academic load with learning stage, the prevalence of astigmatism may also vary at different learning stages. Currently, several studies [7, 12, 15–22] have reported the magnitude of RA and CA, as well as prevalence of RA in children of different ages. However, few studies [15] compared the amount and prevalence of RA and CA among different learning stages in detail.

Furthermore, previous studies have reported risk factors for astigmatism, including ethnicity, age, and spherical equivalent (SE) [15, 23, 24]. Several studies [23, 25] showed that children with SE ≤ − 1.0 D were more likely to have astigmatism, and many studies [26–29] have found that near work is a risk factor for myopia. It is worth noting whether near work is also a risk factor for astigmatism. A study [30] conducted in Singapore demonstrated that the amount of astigmatism might be associated with near viewing behaviors such as playing video games and computers. However, few studies reported related near-work factors for astigmatism, including near work time and near work related behaviors.

Therefore, this study aimed to compare the magnitude and prevalence of RA and CA in Chinese children aged 4–15 years among three learning stages, analyze the age-specific amount and prevalence of astigmatism at each learning stage, and investigate the potential associated factors of near work and outdoor activity time with the two components of astigmatism.

## Methods

### Subjects

This was a school-based, cross-sectional study conducted in Minhang District, Shanghai, China in 2020. A cluster sampling was used. Based on 310 schools in this district, 19 schools including 12 kindergartens, 4 primary schools, and 3 junior high schools were randomly selected. 8858 children aged 4–15 years old from selected schools, including 2694 preschool children, 4077 students from primary school, and 2087 students from junior high school were enrolled in the study, and those who had eye diseases such as corneal opacities, cataract, glaucoma, and retinopathy or had a history of eye injury or eye surgery were excluded. Sample size was based on previous reported prevalence of astigmatism [13, 31, 32], with the tolerable error of 0.025, 0.025 and 0.035, respectively, and with a 95% confidence interval. Assuming a design effect of 1.5 and nonparticipation of 20%, 1850 preschool children, 2300 primary school students, and 1450 junior high school students were required. The Institutional Review Board of the Eye and ENT Hospital of Fudan University approved this study, which was conducted following the principles of the Declaration of Helsinki. All of the children’s parents or guardians provided written informed consent.

### Examinations

Three trained and experienced ophthalmologists conducted the eye examinations. The noncycloplegic refraction of the children’s eyes was measured using an autorefractor (Cannon RF10, Tokyo, Japan). The corneal curvature and axial length were obtained with an ocular biometry system (IOL Master 500, ZEISS, Germany). At least one of each child’s legal guardians together with child were invited to completed a comprehensive questionnaire, and the details about the questionnaire were explained to the parents by the project members. The detailed questionnaire included basic information regarding the children (name, age, native place, birth time, school, class, grade), parental myopia, preterm birth, birth weight, time spent on near work and outdoor activities, and near work related behaviors. Time spent on near work included daily homework completion time on weekdays and weekends (hour/day). Time spent on outdoor activities included daily outdoor activities time on weekdays and weekends (hour/day). Near work related behaviors included continuous near work for more than 30–40 min as well as watching television within two meters (by asking “how often this behavior occurs”, the responses were coded as three options, including “almost none”, “sometimes” and “often”). Before the formal investigation began, the repeatability of the questionnaire was tested by asking 50 parents to complete two same surveys with a two-week interval, and an intraclass correlation of 0.89 was found.

### Definition

The magnitude of RA refers to the cylindrical power, expressed as a negative cylinder form. CA was calculated as the difference between the flattest and steepest meridians power of the anterior corneal surface with the power calculated as (1.3375 − 1)/r, where r refers to the anterior corneal curvature radius, and 1.3375 is the equivalent refractive index value [33]. The cylindrical axis is equal to the flattest meridian. Presence of RA and CA was defined as a cylindrical power ≤ − 1.0 D and the amount of CA ≤ − 1.0 D, respectively.

### Statistical analysis

Statistical analyses were performed using the Statistical Package for Social Sciences (SPSS version 26.0, IBM, Chicago, IL, USA). Categorical and continuous variables were expressed as percentages and the mean ± SD, respectively. Because of the significant correlations in RA and CA between the right eyes and left eyes (Spearman correlation tests, *P* < 0.001), this study used the right eye data for analysis. One-way analysis of variance (ANOVA) and Dunnett’s T3 or Bonferroni test were used to compare astigmatism magnitude across different age groups or learning stages and for post hoc tests, respectively. And the magnitude was also compared between boys and girls using the Manner Whitney U Test. Chi-square test was used to compare the prevalence of RA and CA across different learning stages, and the Bonferroni test was used for post-hoc comparisons. Multivariate linear and logistic regressions were used to analyze the factors related to the amount of RA and CA and to evaluate the risk factors for the presence of RA and CA ( ≤ − 1.00 D), respectively. *P* < 0.05 was considered statistically significant.

## Results

### Participants

Overall, 8858 children aged 4–15 years participated in examinations and received questionnaires, and 7084 children, including 3663 boys (51.7%), completed the examination, with a mean age of 8.08 ± 3.11 years. 2045 children from kindergartens (mean age of 4.64 ± 0.64 years), 3296 children from primary schools (mean age of 7.86 ± 1.40 years), and 1743 students from junior high schools (mean age of 12.54 ± 1.15 years) were enrolled (Table 1).

**Table 1:** The magnitude of RA and CA by different learning stages and gender

Variables	N	RA	^‡^*P* value	CA	^‡^*P* value
		Mean ± SD (D)		Mean ± SD (D)	
Total	7084	−0.61 ± 0.62		−1.20 ± 0.64	
Age(years)					
kindergarten					
Total	2045	−0.53 ± 0.53^a^		−1.14 ± 0.63^a^	
4	911	−0.55 ± 0.56	0.132	−1.17 ± 0.64	0.049
5	957	−0.50 ± 0.49		−1.10 ± 0.62	
6	177	−0.55 ± 0.59		−1.12 ± 0.61	
Primary school					
Total	3296	−0.56 ± 0.57^a^		−1.16 ± 0.62^a^	
6	641	−0.52 ± 0.56	<0.001	−1.14 ± 0.60	0.009
7	851	−0.51 ± 0.52		−1.13 ± 0.62	
8	752	−0.52 ± 0.51		−1.17 ± 0.66	
9	506	−0.59 ± 0.61		−1.20 ± 0.60	
10	463	−0.69 ± 0.68		−1.25 ± 0.62	
11	83	−0.57 ± 0.44		−1.10 ± 0.46	
Junior high school					
Total	1743	−0.81 ± 0.76^b^		−1.34 ± 0.67^b^	
11	384	−0.76 ± 0.75	0.132	−1.34 ± 0.70	0.354
12	512	−0.77 ± 0.69		−1.31 ± 0.62	
13	448	−0.85 ± 0.81		−1.36 ± 0.68	
14	326	−0.88 ± 0.83		−1.37 ± 0.69	
15	73	−0.77 ± 0.64		−1.23 ± 0.65	
^†^*P* value	**<0.001**			**<0.001**	
Gender					
Boy	3663	−0.62 ± 0.63	0.015	−1.18 ± 0.66	<0.001
Girl	3421	−0.59 ± 0.61		−1.22 ± 0.61	

RA, refractive astigmatism; CA, corneal astigmatism; ^†^*P*, one-way ANOVA comparing RA and CA values across the three learning stages; ^a^ and ^b^, statistical difference in the post hoc comparisons; ^‡^*P*, one-way ANOVA comparing RA and CA values across different age groups within different learning stages or Manner Whitney U Test comparing RA and CA values between boys and girls

### The magnitude of RA and CA

In this study, the mean amount of RA and CA in all children aged 4–15 years was − 0.61 ± 0.62 D and − 1.20 ± 0.64D, respectively. Significant differences in the mean RA and CA values were observed among the three learning stages (both *P* < 0.001). The post-hoc tests showed that the amount of RA and CA in the junior high school children were the highest when compared with that in kindergarten (both *P* < 0.001) and primary school (both *P* < 0.001), respectively (Table 1), while no statistical difference was observed between kindergarten and primary school (both *P* > 0.05).

**Fig. 1:**
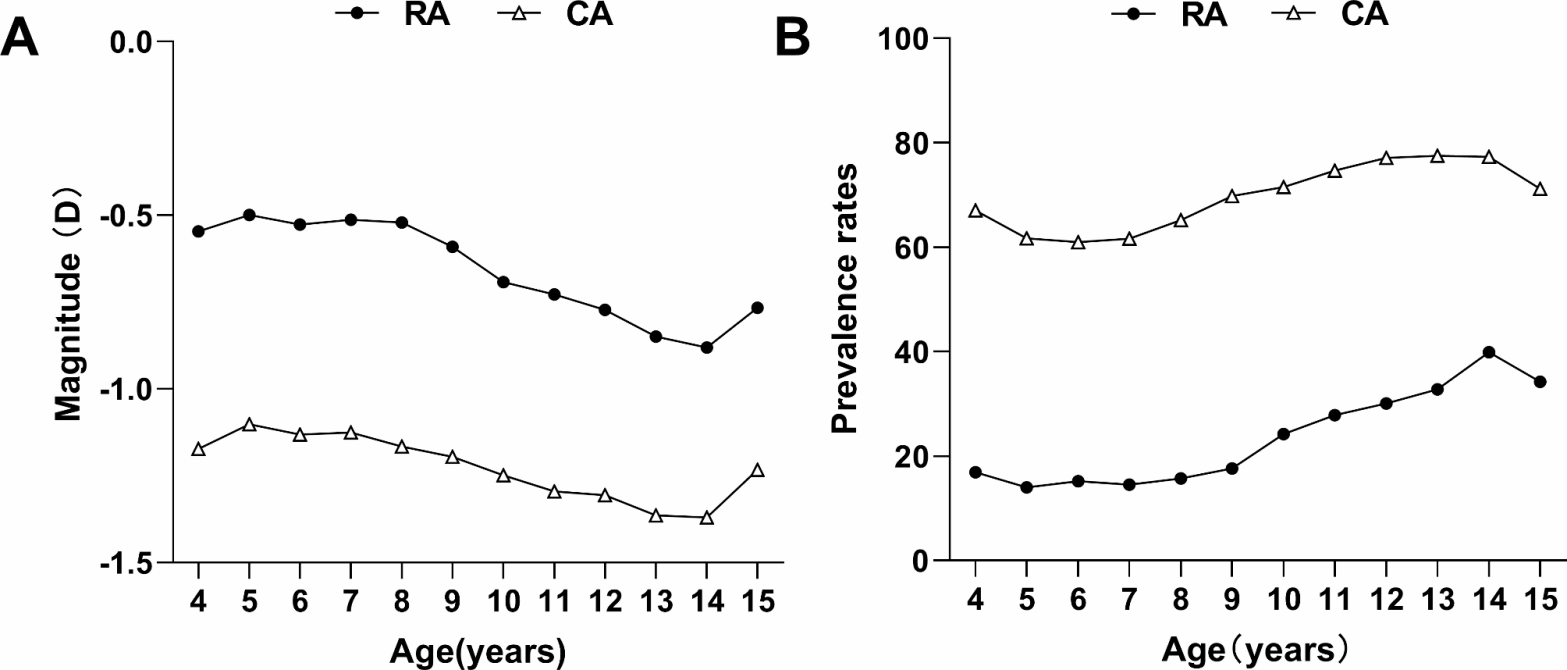
The magnitude and prevalence of RA and CA changed with age. RA, refractive astigmatism; CA, corneal astigmatism

The magnitude of RA and CA generally increased with the advancement of learning stages (One-way ANOVA, *P* < 0.001) and with age from 4 to 15 years (*P* trend < 0.001) (Fig. 1, Supplementary Table S1), while the RA and CA values varied with age differently within different learning stages. In the primary school, the amount of RA and CA increased with age (Table 1) (One-way ANOVA, both *P* < 0.01). In contrast, at kindergarten and junior high school stages, there was no significant difference in RA and CA across different ages.

**Fig. 2:**
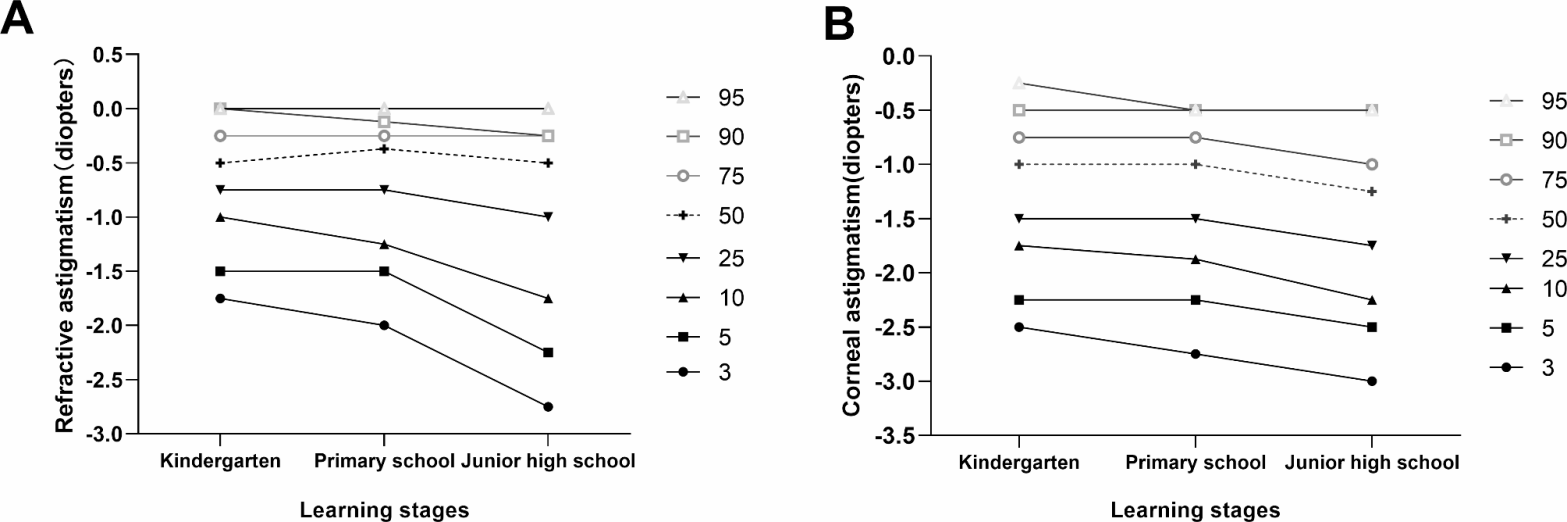
Percentiles distribution (The 3rd, 5th, 10th, 25th, 50th, 75th, 90th and 95th percentiles) of RA and CA values based on the learning stages. RA, refractive astigmatism; CA, corneal astigmatism

Figure 2 shows percentiles of the RA and CA values based on the learning stages. The spread of RA increased with the advancement of learning stage, from the RA range in kindergarten (between the 3rd and 95th percentile) of 1.75 D to the RA range in junior high school of 2.75 D. Although the CA range (between the 3rd and 95th centile) also increased with the learning stages, the increase was slight (0.25D).

A higher magnitude of RA was associated with older age (standardized coefficient beta = − 0.167, *P* < 0.001), higher myopic spherical power (beta = 0.033, *P* = 0.019), steeper corneal curvature (beta = − 0.128, *P* < 0.001), boy (beta = 0.059, *P* < 0.001), and paternal myopia (beta = − 0.026, *P* = 0.032). In addition, a higher magnitude of CA was associated with older age (beta = − 0.167, *P* < 0.001), higher hyperopic spherical power (beta = − 0.054, *P* < 0.001), and the steeper corneal curvature (beta = − 0.213, *P* < 0.001) (Table 2).

**Table 2:** Multivariate linear regression analysis of the factors with magnitude of RA and CA

Factors	RA (D)				CA (D)		
	Standardized Coefficient Beta	t	*P* value		Standardized Coefficient Beta	t	*P* value
Age (years)	−**0.167**	−**9.656**	**< 0.001**		**−0.159**	**-9.216**	**< 0.001**
Spherical power (D)	**0.033**	**2.344**	**0.019**		**−0.054**	**-3.869**	**< 0.001**
Mean corneal curvature (D)	−**0.128**	−**10.594**	**< 0.001**		**−0.213**	**-17.677**	**< 0.001**
Gender							
Boy	Reference				Reference		
Girl	**0.059**	**4.891**	**< 0.001**		0.022	1.837	0.066
Paternal myopia							
No	Reference				Reference		
Yes	**−0.026**	**−2.145**	**0.032**		−0.005	-0.419	0.675
Maternal myopia							
No	Reference				Reference		
Yes	−0.02	−1.582	0.114		−0.022	−1.771	0.077
Preterm birth							
No	Reference				Reference		
Yes	−0.002	−0.196	0.845		−0.014	−1.126	0.26
Watching TV within two meters							
Almost none	Reference				Reference		
Sometimes	−0.004	−0.373	0.709		−0.021	−1.743	0.081
Often	−0.007	−0.619	0.536		0.005	0.43	0.667
Continuous near work for more than 30–40 min							
Almost none	Reference				Reference		
Sometimes	−0.002	−0.114	0.91		−0.001	−0.085	0.932
Often	0.02	1.255	0.209		0.008	0.516	0.606
Homework time on weekdays (hour/day)	0.008	0.486	0.627		0.005	0.3	0.765
Homework time on weekends (hour/day)	−0.009	−0.551	0.582		−0.007	−0.402	0.687
Outdoor activity time on weekdays (hour/day)	0.001	0.105	0.916		−0.002	−0.165	0.869
Outdoor activity time on weekends (hour/day)	0.022	1.696	0.09		0.019	1.449	0.147
Birth weight (g)	−0.002	−0.196	0.845		−0.013	−1.093	0.274

RA, refractive astigmatism; CA, corneal astigmatism

### Prevalence of RA and CA

The overall prevalence of RA and CA in children aged 4–15 years was 20.3% (95% confidence interval (CI): 19.4–21.2%) and 67.6% (95% CI: 66.5–68.8%), respectively. Across the three learning stages, significant differences in the prevalence of RA and CA (≤ − 1.0D) were found (both *P* < 0.001), with the highest prevalence in junior high school (RA, 32.8%; CA, 76.9%) (Table 3). In addition, post-hoc comparisons found no statistical difference in the prevalence of RA and CA between kindergarten and primary school. The prevalence of RA in boys was higher than that in girls (21.8% vs. 18.8%, *P* = 0.002), while the prevalence of CA in boys was lower than girls (65.3% vs. 70.2%, *P* < 0.001).

**Table 3:** Prevalence of RA and CA by different learning stages and gender

Variables	N	RA		^‡^*P* value	CA		^‡^*P* value
		N	% (95% CI)		N	% (95% CI)	
Total		1440	20.3 (19.3–21.3)		4792	67.6 (66.6–68.7)	
Age(year)							
kindergarten							
Total	2045	324	15.8 (14.3–17.4)^a^		1309	64 (61.9–66.3)^a^	
4	911	154	16.9 (14.5–19.4)	0.053	610	67.0 (64–70)	0.045
5	957	134	14.0 (11.9–16.1)		590	61.7 (58.6–64.8)	
6	177	36	20.3 (14.7–26.6)		109	61.6 (53.7–68.9)	
Primary school							
Total	3296	545	16.5 (15.2–17.8)^a^		2142	65 (63.4–66.7)^a^	
6	641	88	13.7 (11.1–16.5)	< 0.001	390	60.8 (56.8–64.6)	< 0.001
7	851	123	14.5 (12.1–16.8)		524	61.6 (58.2–64.9)	
8	752	118	15.7 (13.2–18.5)		490	65.2 (62.0–68.5)	
9	506	89	17.6 (14.2–20.9)		353	69.8 (65.8–73.3)	
10	463	112	24.2 (20.5–28.1)		331	71.5 (67.4–75.6)	
11	83	15	18.1 (9.6–27.7)		54	65.1 (54.2–74.7)	
Junior high school							
Total	1743	571	32.8 (30.5–34.7)^b^		1341	76.9 (74.9–78.8)^b^	
11	384	115	29.9 (25.3–34.6)	0.031	295	76.8 (72.9–81.0)	0.836
12	512	154	30.1 (26.0–34.0)		395	77.1 (73.6–80.7)	
13	448	147	32.8 (28.6–37.3)		347	77.5 (73.4–81.0)	
14	326	130	39.9 (35.0–45.1)		252	77.3 (73.0–81.9)	
15	73	25	34.2 (23.3–45.2)		52	71.2 (61.6–80.8)	
^†^*P* value			**<0.001**			**<0.001**	
Gender							
Boy	3663	797	21.8 (20.3–23.1)	0.002	2392	65.3 (63.7–66.9)	0.001
Girl	3421	643	18.8 (17.5–20.2)		2400	70.2 (68.6–71.7)	

RA, refractive astigmatism; CA, corneal astigmatism;^†^*P*, the chi-square test comparing the prevalence of RA and CA across three learning stages; ^a^ and ^b^, statistical difference in post hoc comparisons; ^‡^*P*, the chi-square test comparing the prevalence of RA and CA across different age groups within various learning stages and between boys and girls

The RA and CA prevalence rates increased with age in the entire age range (Chi-square test, both *P* trend < 0.001) (Fig. 1), but varied with age differently within various learning stages (Table 3). At the kindergarten stage, chi-square test and post-hoc comparisons showed no significant difference in RA and CA across various age groups. At the primary school stage, the prevalence of RA and CA increased with age. However, at the junior high school stage, the prevalence of RA was different across age groups, whereas no difference in CA prevalence was detected across age groups.

**Fig. 3:**
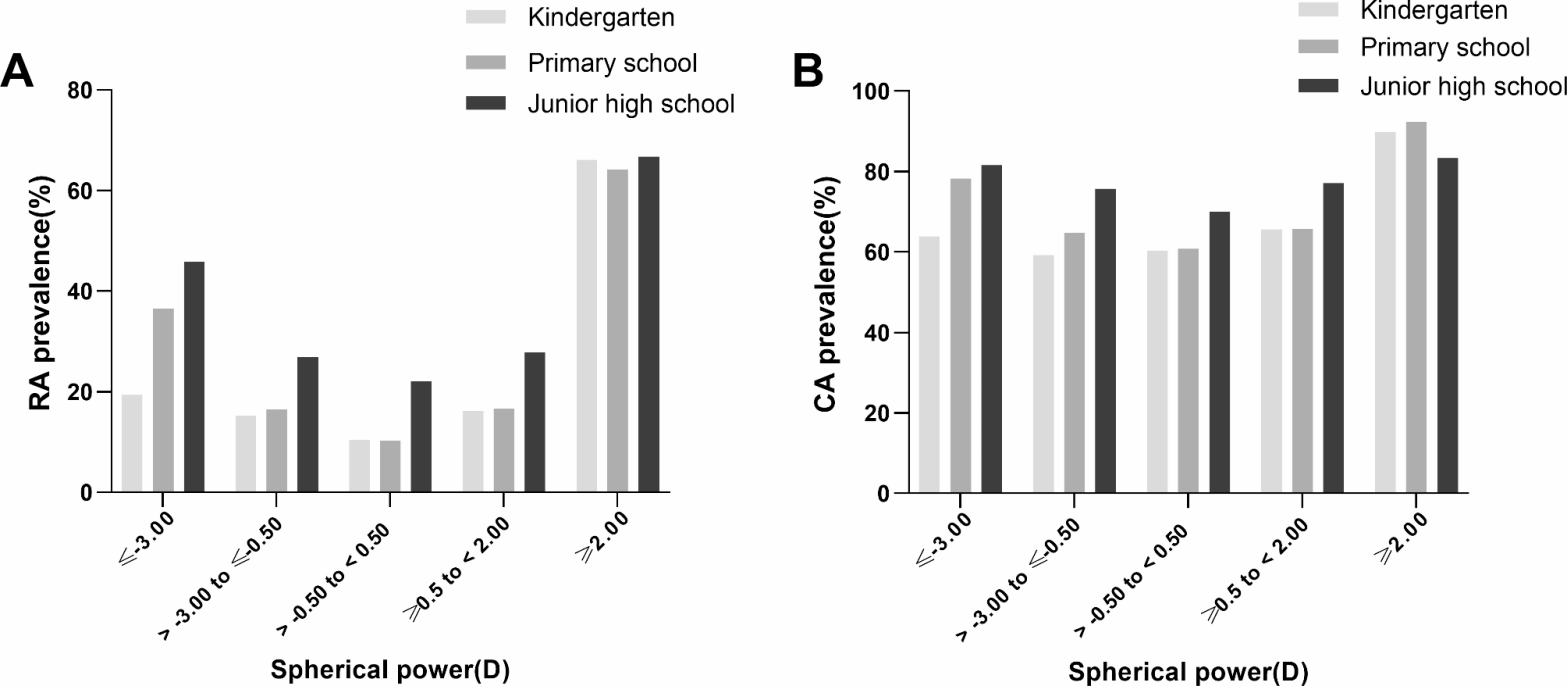
Prevalence of RA and CA at each learning stage by different spherical power groups. RA, refractive astigmatism; CA, corneal astigmatism

Figure 3 shows the prevalence of RA and CA at different learning stages classified by spherical power. The overall prevalence of RA and CA was the lowest when the spherical power was between − 0.5 and 0.5 D (RA, 11.8%, 95% CI, 10.4–13.3%; CA, 61.8%, 95% CI, 59.6–63.8%), and the prevalence at each stage as well as overall prevalence increased with the increasing amount of spherical power (more myopic or hyperopic) (*P* < 0.001) (Supplementary Table S2 and S3).

In this study, boy (OR = 0.698; 95% CI, 0.617–0.790; *P* < 0.001), junior high school (OR = 1.973; 95% CI, 1.579–2.464; *P* < 0.001), steeper mean corneal curvature (OR = 1.24; 95% CI, 1.188–1.295; *P* < 0.001), longer homework time on weekdays (OR = 1.074; 95% CI, 1.007–1.145; *P* = 0.029), and shorter outdoor activities time on weekends (OR = 0.929; 95% CI, 0.877–0.984; *P* = 0.013) were associated with the presence of RA. In addition, the presence of CA was associated with steeper mean corneal curvature (OR = 1.286; 95% CI, 1.239–1.334; *P* < 0.001) and junior high school (OR = 1.828; 95% CI, 1.493–2.238; *P* < 0.001) (Table 4).

**Table 4:** Multivariate logistic analysis of the risk factors for presence of RA and CA

Factors	RA				CA		
	*P* value	OR	95% CI		*P* value	OR	95% CI
Mean corneal curvature (D)	**< 0.001**	**1.24**	**1.188–1.295**		**< 0.001**	**1.286**	**1.239 − 1.334**
Learning stages							
kindergarten	Reference				Reference		
Primary school	0.204	0.893	0.751–1.063		0.885	0.99	0.861 − 1.138
Junior high school	**< 0.001**	**1.973**	**1.579–2.464**		**< 0.001**	**1.828**	**1.493 − 2.238**
Spherical power (D)	**0.021**	**0.956**	**0.921–0.993**		0.744	1.006	0.971 − 1.042
Gender							
Boy	Reference				Reference		
Girl	**< 0.001**	**0.698**	**0.617–0.790**		0.283	1.059	0.954 − 1.177
Homework time on weekdays (hour/day)	**0.029**	**1.074**	**1.007–1.145**		0.06	1.062	0.997 − 1.130
Outdoor activity time on weekends (hour/day)	**0.013**	**0.929**	**0.877–0.984**		0.444	0.983	0.940 − 1.027
Watching TV within two meters							
Almost none	Reference				Reference		
Sometimes	0.126	1.103	0.973–1.250		0.427	1.044	0.938 − 1.162
Often	0.06	1.254	0.991–1.586		0.712	1.041	0.843 − 1.285
Homework time on weekends (hour/day)	0.666	1.01	0.967–1.054		0.808	0.995	0.955 − 1.037
Outdoor activity time on weekdays (hour/day)	0.971	1.002	0.904–1.111		0.815	1.01	0.926 − 1.103
Continuous near work for more than 30–40 min							
Almost none	Reference				Reference		-
Sometimes	0.569	1.048	0.892–1.231		0.708	0.973	0.844 − 1.122
Often	0.235	0.892	0.74–1.077		0.448	0.94	0.803 − 1.102
Birth weight(g)	0.841	1	1.000–1.000		0.398	1	1.000 − 1.000
Preterm birth							
No	Reference				Reference		
Yes	0.642	1.066	0.814–1.396		0.77	1.037	0.813 − 1.322
Paternal myopia							
No	Reference				Reference		
Yes	0.05	1.137	1.000–1.293		0.62	0.973	0.872 − 1.085
Maternal myopia							
No	Reference				Reference		
Yes	0.271	1.076	0.945–1.225		0.566	1.033	0.925 − 1.154

RA, refractive astigmatism; CA, corneal astigmatism

## Discussion

This school-based study evaluated the magnitude and prevalence of RA and CA in a relatively wide age range of children. The magnitudes of astigmatism varied across different regions. Compared with similar ages, the magnitude of RA in this study was slightly higher than that in preschool children (–0.43 D) [34] in Nanjing, China and Australian children [20] aged 6–7 years (–0.29 D). However, it was slightly lower than that in Tohono O’odham Native American children [35] aged 3–11 years (1.26 D). In addition, the magnitude of CA was lower than that of Tohono O’odham Native American children (3–11 years old, 1.85 D) [35] but higher than Australian children(6–7 years, − 0.82D) [20].

The current study found that in a cross-sectional distribution, the magnitudes of RA and CA increased with learning stage in the 4- to 15-year range, and the RA/CA values were highest at the junior high school. The range of RA also expanded with learning stage. Several findings [15, 19, 21, 36] reported the amount of RA and CA increased with age, while few studies directly compared the amount of astigmatism among learning stages.

At different learning stages, the magnitudes of RA and CA displayed divergent trends with age. The amount of RA and CA in the preschool period of 4–6 years was relatively stable with age, which is similar to other studies [37, 38]. while a longitudinal study [34] showed that children aged 4–7 years have an increase in RA of 0.07 D and CA of 0.04 D annually. In this study, the increasing trend with age mainly started at the primary school stage. However, one study [35] showed that children aged 3 and < 11 years old have a reduction of 0.02 D and 0.03 D annually in RA and CA, respectively. Therefore, more longitudinal studies are required to explore age-related changes in RA and CA at different learning stages.

The increases in RA and CA magnitude values with learning stage may be related to the development of children’s eyeballs and the environmental factors such as education. The influence of environmental factors might be minimal at preschool stage, and may be greater with the advancement of learning stage. The magnitudes of RA and CA were stable with age in kindergarten and junior high school, but increased with age at primary school stage, which implies that the primary school stage might be a crucial period for development of children’s refraction and ocular biometrics. Therefore, prevention and control of myopia and astigmatism should be noticed during and before this stage [39].

In the current study, a higher amount of RA was associated with older age, more negative spherical power, greater corneal curvature, male gender, which is consistent with other studies [15, 36]. Moreover, paternal myopia was also found to be associated with the higher amount of RA, although it is usually considered as a risk factor for myopia.

The prevalence of astigmatism also varies significantly with region and ethnicity. The RA prevalence rate in this study was lower than children with similar age in Northern Ireland (6–7 years of age, 24%) [12], America (Native Tohono O’odham tribe, 5–16 years of age, 34.7%) [18], and Wuxi, China (3–6 years, 36%) [40]. However, it was relatively higher than that in Australia (6 years of age, 4.8%; 12 years of age, 6.7%) [10, 20] and Nanjing, China (4–5 years of age, 14.2%).

The CA prevalence rate was relatively high with more than 50% at each learning stage. It was higher than that in Australia (6 years of age, 27.7%; 12 years of age, 26.6%) [10, 20], and Anyang City, China (12 years of age, 52.8%) [7], but it was lower than Tohono O’odham Native American children aged 6 months to 8 years (78.3%) [21].

Similar to the changes in the magnitude of RA, the prevalence of RA also increased with the learning stage from preschool to school. In detail, the phenomenon that RA prevalence increased with age mainly occurred at the primary school and junior high school stage, which is similar to other findings [15, 19, 36, 41]. However, several studies have demonstrated that the prevalence of RA in the school-age stage was stable with age [10, 12, 18], or decreased with age [42, 43]. Some studies showed that the RA prevalence decreased with age from infant stage to early childhood [44–46], while the current study showed that the prevalence of RA in kindergarten was stable with age, which is consistent with the results of some studies [9, 17, 37]. Therefore, it can be assumed that RA prevalence might decrease from infancy to young children stage and stabilize during the preschool stage. Moreover, further longitude studies are required to investigate the change in RA prevalence with age at the school-age stage.

In this study, no significant change of CA prevalence with age was observed in preschool stage, which is consistent with the study in America [21] and Nanjing, China [33]. In contrast, at the primary school stage, the prevalence of CA increased with age. Currently, few studies have reported the prevalence of CA increasing with age in the school-age children. Moreover, some studies reported no differences in the prevalence of CA between 6 and 7 and 12–13 years old children [12] in Northern Ireland, and between 6 and 12 years old children [10] in Australia.

This study evaluated the prevalence of astigmatism based on the spherical power. It was found that in general, when the spherical power was within the emmetropic range (-0.5–0.5 D), the prevalence of RA and CA was the lowest. Furthermore, the more myopic or hyperopic the spherical power, the higher the RA/CA prevalence, which is similar to the results of other studies [7, 10, 15, 25].

Previous studies have reported that some risk factors for astigmatism include age, myopia, hyperopia, gender, region, and axial length/corneal radius ratio (AL/CR) [15, 24, 25]; however, few studies [40] demonstrated the relationship between RA and time spent on near work and outdoor activities. In the current study, longer daily homework time on weekdays, shorter outdoor sports time on weekends, and male gender were associated with the presence of RA (≤ − 1.0 D). It is worth noting that there were few studies having reported that the occurrence of astigmatism was related to near work time. In addition, shorter daily outdoor sports time on weekends might also be a risk factor for RA, which is consistent with a study [47] that showed children with astigmatism engaged in fewer outdoor activities than their peers without astigmatism. These results imply that time spent on near work and outdoor activities not only affect myopia, but also affect astigmatism. Therefore, paying attention to near work and outdoor activities is crucial. In this study, male was also one of the risk factors for RA, which is similar to a previous study [15], but other studies [30, 33] showed that RA was unrelated to gender. More longitudinal research is needed to explore the relationship between near work time, gender and RA.

In current study, the presence of CA (≤ − 1.0 D) was associated only with steeper mean corneal curvature and junior high school stage. A study reported [33] no correlation between CA and age, gender, and AL/CR, which is similar to the results in this study; however this study [33] showed that larger AL/CR, work during pregnancy, and cesarean section might be risk factors for anterior CA. Therefore, further studies focusing on the risk factors for CA are required.

The strengths of this study lie in the large school-based population and the wide age range (4–15 years of age). In addition, potential associations of time spent on near work and outdoor activities with RA were found in this study. This research had some limitations. First, this study used noncycloplegic autorefraction, which may have an impact on RA, to a certain extent. However, some studies [48, 49] have shown that no difference is found in RA between noncycloplegic and cycloplegic autorefraction measurements. Second, this study provided cross-sectional results. Findings about relationship between astigmatism and near work from longitudinal studies would be more convincing. Therefore, further longitudinal studies are required to explore the association between astigmatism and near work related factors.

## Conclusion

In summary, this study found that the magnitude and prevalence of RA and CA increased with age in a wide age range and with the advancement of the learning stage, from kindergarten to junior high school. These increases mainly began at the primary school stage. In addition, the prevalence of CA was relatively high, with all higher than 50% in the three learning stages. Longer homework time on weekdays and shorter outdoor activities time on weekends were associated with the presence of RA.

### Electronic supplementary material

Below is the link to the electronic supplementary material.

Supplementary Material 1

Supplementary Material 2

Supplementary Material 3

Supplementary Material 4

## Data Availability

The data analyzed to support the findings of this study are available from the corresponding author on reasonable request.

## Abbreviations

RA: Refractive Astigmatism
CA: Corneal Astigmatism
SD: Standard Deviation
OR: Odds Ratio
SE: Spherical Equivalent
ANOVA: One-way Analysis of Variance
CI: Confidence Interval
