# Supplementary material for: Refractive and corneal astigmatism in Chinese 4–15 years old children: prevalence and risk factors
Source: BMC Ophthalmol. 2023 Nov 10;23:449. doi: 10.1186/s12886-023-03201-y (PMC10638796; doi:10.1186/s12886-023-03201-y)
Supplement: Supplementary file 1 — Supplementary Material 1 [file 12886_2023_3201_MOESM1_ESM.docx]

Table S1. The magnitude and prevalence of RA and CA by age groups

| Age (years) | RA | | CA | |
| --- | --- | --- | --- | --- |
|  | Mean (SD) | % (N) | Mean (SD) | % (N) |
| 4 | −0.55 (0.56) | 16.9 (154) | −1.17 (0.64) | 67.0 (610) |
| 5 | −0.50 (0.49) | 14.0 (134) | −1.10 (0.62) | 61.7 (590) |
| 6 | −0.53 (0.57) | 15.2 (124) | −1.13 (0.61) | 61.0 (499) |
| 7 | −0.51 (0.52) | 14.5 (123) | −1.13 (0.62) | 61.6 (524) |
| 8 | −0.52 (0.51) | 15.7 (118) | −1.17 (0.66) | 65.2 (490) |
| 9 | −0.59 (0.61) | 17.6 (89) | −1.20 (0.60) | 69.8 (353) |
| 10 | −0.69 (0.68) | 24.2 (112) | −1.25 (0.62) | 71.5 (331) |
| 11 | −0.73 (0.71) | 27.8 (130) | −1.30 (0.67) | 74.7 (349) |
| 12 | −0.77 (0.69) | 30.1 (154) | −1.31 (0.62) | 77.1 (395) |
| 13 | −0.85 (0.81) | 32.8 (147) | −1.36 (0.68) | 77.5 (347) |
| 14 | −0.88 (0.83) | 39.9 (130) | −1.37 (0.69) | 77.3 (252) |
| 15 | −0.77 (0.64) | 34.2 (25) | −1.23 (0.65) | 71.2 (52） |
| ^†^*P* value | <0.001 | <0.001 | <0.001 | <0.001 |

RA, refractive astigmatism; CA, corneal astigmatism; ^†^*P,* one-way ANOVA and chi-square test for comparing the magnitude and prevalence of RA and CA across age groups, respectively.
