# Supplementary material for: Refractive and corneal astigmatism in Chinese 4–15 years old children: prevalence and risk factors
Source: BMC Ophthalmol. 2023 Nov 10;23:449. doi: 10.1186/s12886-023-03201-y (PMC10638796; doi:10.1186/s12886-023-03201-y)
Supplement: Supplementary file 2 — Supplementary Material 2 [file 12886_2023_3201_MOESM2_ESM.docx]

Table S2. Prevalence of RA by spherical power

| Variables | spherical power ≤−3.0D | | −3<spherical power≤−0.5D | | −0.5<spherical power<0.5D | | 0.5≤spherical power<2.0D | | spherical power ≥2.0D | | ^‡^*P* value |
| --- | --- | --- | --- | --- | --- | --- | --- | --- | --- | --- | --- |
|  | % (N) | 95% CI | % (N) | 95% CI | % (N) | 95% CI | % (N) | 95% CI | % (N) | 95% CI |  |
| Kindergarten | 19.4%(7) | 8.3−33.3 | 15.2%(34) | 10.8−20.2 | 10.4%(63) | 7.9−12.9 | 16.1%(181) | 14.1−18.3 | 66.1%(39) | 52.5−78.0 | <0.001 |
| Primary school | 36.5%(57) | 28.8−44.2 | 16.5%(158) | 14.3−18.9 | 10.2%(112) | 8.5−12.1 | 16.6%(168) | 14.6−19.0 | 64.1%(50) | 53.8−74.4 | <0.001 |
| Junior high school | 45.8%(251) | 42.0−50.2 | 26.9%(209) | 23.6−30.1 | 22%(55) | 16.8−27.6 | 27.8%(40) | 20.8−35.4 | 66.7%(16) | 45.9−83.3 | <0.001 |
| ^†^*P* value | <0.001 |  | 0.002 |  | <0.001 |  | 0.002 |  | 0.958 |  |  |
| Boy | 45.5%(162) | 40.4−50.6 | 23.1%(226) | 20.3−26.0 | 12.8%(132) | 10.7−14.9 | 18.6%(228) | 16.4−20.8 | 71%(49) | 60.9−81.2 | <0.001 |
| Girl | 39.8%(153) | 34.6−44.5 | 17.9%(175) | 15.4−20.3 | 10.7%(98) | 8.8−12.8 | 15.3%(161) | 13.2−17.5 | 60.9%(56) | 51.1−70.7 | <0.001 |
| ^†^*P* value | 0.12 |  | 0.004 |  | 0.171 |  | 0.033 |  | 0.181 |  |  |
| Total | 42.6%(315) | 39.1−45.9 | 20.5%(401) | 18.6−22.3 | 11.8%(230) | 10.4−13.3 | 17.1%(389) | 15.5−18.7 | 65.2%(105) | 57.8−72.7 | <0.001 |

RA, refractive astigmatism; CA, corneal astigmatism; ^†^*P*, chi-square test for comparing the prevalence across different learning stages and between boys and girls; ^‡^*P*, chi-square test for comparing the prevalence across different spherical power groups
