# Supplementary material for: Refractive and corneal astigmatism in Chinese 4–15 years old children: prevalence and risk factors
Source: BMC Ophthalmol. 2023 Nov 10;23:449. doi: 10.1186/s12886-023-03201-y (PMC10638796; doi:10.1186/s12886-023-03201-y)
Supplement: Supplementary file 3 — Supplementary Material 3 [file 12886_2023_3201_MOESM3_ESM.docx]

Table S3. Prevalence of CA by spherical power

| Variables | spherical power ≤-3.0D | | -3<spherical power≤-0.5 | | | -0.5<spherical power<0.5 | | 0.5≤spherical power<2.0 | | spherical power≥2.0 | | ^‡^*P* value |
| --- | --- | --- | --- | --- | --- | --- | --- | --- | --- | --- | --- | --- |
|  | % (N) | 95% CI | % (N) | 95% CI | | %(N) | 95% CI | % (N) | 95% CI | % (N) | 95% CI |  |
| Kindergarten | 63.9%(23) | 47.2−80.6 | 59.2%(132) | 52.9−65.9 | | 60.3%(364) | 56.5−64.1 | 65.6%(737) | 63.0−68.4 | 89.8%(53) | 83.1−96.6 | <0.001 |
| Primary school | 78.2%(122) | 71.2−84.6 | 64.7%(620) | 61.6−67.9 | | 60.8%(664) | 57.8−63.7 | 65.7%(664) | 62.7−68.7 | 92.3%(72) | 85.9−97.4 | <0.001 |
| Junior high school | 81.6%(447) | 78.3−84.7 | 75.7%(588) | 72.7−78.8 | | 70.0%(175) | 64.0−76.0 | 77.1%(111) | 69.4−84.0 | 83.3%(20) | 66.7−95.8 | 0.005 |
| ^†^*P* value | 0.03 |  | <0.001 |  | | 0.016 |  | 0.02 |  | 0.389 |  |  |
| Boy | 80.6%(287) | 76.4−84.6 | 64.3%(630) | 61.2−67.4 | | 59.5%(616) | 56.5−62.1 | 65.4%(800) | 62.6−68.1 | 85.5%(59) | 76.8−92.8 | <0.001 |
| Girl | 79.4%(305) | 75.0−83.6 | 72.5%(710) | 69.9−75.2 | | 64.4%(587) | 61.4−67.5 | 67.6%(712) | 64.5−70.4 | 93.5%(86) | 88.0−97.8 | <0.001 |
| ^†^*P* value | 0.686 |  | <0.001 |  | | 0.028 |  | 0.281 |  | 0.094 |  |  |
| Total | 80.0%(592) | 77.0−82.8 | 68.4%(1340) | | 66.4−70.5 | 61.8%(1203) | 59.6−63.8 | 66.4%(1512) | 64.3−68.3 | 90.1%(145) | 85.7−94.4 | <0.001 |

RA, refractive astigmatism; CA, corneal astigmatism; ^†^*P*, chi-square test for comparing the prevalence across different learning stages and between boys and girls; ^‡^*P*, chi-square test for comparing the prevalence across different spherical power group.
