# Supplementary material for: Refractive and corneal astigmatism in Chinese 4–15 years old children: prevalence and risk factors
Source: BMC Ophthalmol. 2023 Nov 10;23:449. doi: 10.1186/s12886-023-03201-y (PMC10638796; doi:10.1186/s12886-023-03201-y)
Supplement: Supplementary file 4 — Supplementary Material 4 [file 12886_2023_3201_MOESM4_ESM.docx]

**Basic information**

Date:__ ____ ____

| Children Name |  | ID number |  |
| --- | --- | --- | --- |
| Gender |  | Date of birth |  |
| Native place |  | School |  |
| Grade |  | Class |  |
| Written by children and | □Mother □Father  □Others_____ | Parents’ phone number |  |

**Related factors**

| Preterm birth (Infants are born preterm at less than 37 weeks' gestational age ): | □ Yes □ Not |
| --- | --- |
| Paternal myopia: | □ Yes □ Not |
| Maternal myopia: | □ Yes □ Not |
| Continuous near work for more than 30-40 minutes: | □Almost none □Sometimes □Often |
| Watching TV within two meters: | □Almost none □Sometimes □Often |
|  | |
| Birth weight: | _____g |
| Homework (paper work for exercises or tutoring, reading books) time on weekdays: | _____hours _____minutes (average time per day in this semester) |
| Homework (paper work for exercises or tutoring, reading books) time on weekends: | _____hours _____minutes (average time per day in this semester) |
| Outdoor activity (walking, playing outdoors, having picnics, and playing outdoor sports and so on) time on weekdays | _____hours _____minutes (average time per day in this semester) |
| Outdoor activity (walking, playing outdoors, having picnics, and playing outdoor sports and so on) time on weekends | _____hours _____minutes (average time per day in this semester) |
